# Exosomal miRNAs in tumor microenvironment

**DOI:** 10.1186/s13046-020-01570-6

**Published:** 2020-04-16

**Authors:** Shiming Tan, Longzheng Xia, Pin Yi, Yaqian Han, Lu Tang, Qing Pan, Yutong Tian, Shan Rao, Linda Oyang, Jiaxin Liang, Jinguan Lin, Min Su, Yingrui Shi, Deliang Cao, Yujuan Zhou, Qianjin Liao

**Affiliations:** 1 Hunan Key Laboratory of Translational Radiation Oncology, Hunan Cancer Hospital and The Affiliated Cancer Hospital of Xiangya School of Medicine, Central South University, 283 Tongzipo Road, Changsha, 410013 Hunan China; 2 University of South China, Hengyang, 421001 Hunan China; 3 Department of Medical Microbiology, Immunology & Cell Biology, Simmons Cancer Institute, Southern Illinois University School of Medicine, 913 N. Rutledge Street, Springfield, IL 62794, USA

**Keywords:** exosomal miRNAs, tumor microenvironment (TME), CAFs, angiogenesis, immune microenvironment

## Abstract

Tumor microenvironment (TME) is the internal environment in which tumor cells survive, consisting of tumor cells, fibroblasts, endothelial cells, and immune cells, as well as non-cellular components, such as exosomes and cytokines. Exosomes are tiny extracellular vesicles (40-160nm) containing active substances, such as proteins, lipids and nucleic acids. Exosomes carry biologically active miRNAs to shuttle between tumor cells and TME, thereby affecting tumor development. Tumor-derived exosomal miRNAs induce matrix reprogramming in TME, creating a microenvironment that is conducive to tumor growth, metastasis, immune escape and chemotherapy resistance. In this review, we updated the role of exosomal miRNAs in the process of TME reshaping.

## Background

TME is a complex ecosystem and an important player in all stages of tumorigenesis. TME consists of cancer cells, cancer-associated fibroblasts (CAFs), endothelial cells, immune cells, extracellular matrix (ECM), microvessels, and biomolecules infiltrated [1–5]. Compared with the normal internal environment, TME is more prominently characterized by hypoxia. Hypoxia caused by rapid appreciation of tumor cells leads to release of matrix metalloproteinases (MMPs), hypoxia inducible factor-1α (HIF-1α), vascular endothelial growth factor (VEGF) and other stimulating factors. Reshaping TME provides a niche for interaction between tumor cells and surrounding fibroblasts, endothelial cells, and immune cells [4, 6–9]. These cells interact with tumor cells through TME to induce a variety of biological events, such as appreciation, migration, angiogenesis, immunosuppression, and drug resistance for tumor development [10–14].

MicroRNAs (miRNAs) are a class of short ncRNAs with 19-25 nucleotides in length [15]. Through regulating gene expression, miRNAs regulate a variety of important biological functions, such as proliferation, apoptosis, differentiation, migration, invasion and drug resistance. Genetic or epigenetic changes in cancer cells can induce abnormal expression of miRNAs, thus causing abnormal expression of their target genes [16–21]. miRNAs function through 6-7 base complementary binding to target mRNA and inhibition of target gene expression at the level of protein [22–24]. From the literature, miRNAs can work as oncogenes to promote the formation and biological changes of TME [25–28]. For example, miR-9 and miR-200s induce normal fibroblasts (NFs) in TME to transform into CAFs and promote tumor metastasis [29, 30], miR-526b and miR-655 promote angiogenesis and lymphangiogenesis in TME [31], and miR-340-5p and miR-561 induce formation of immunosuppressive microenvironment [32, 33]. How these biologically active miRNAs are transmitted and function in cells and TME is an important breakthrough in the study of TME.

Recently, exosomes are considered to be the key mediators responsible for the heterogeneity of the TME and carry biologically active cargos, such as protein, metabolites, nucleic acids (e.g. ncRNAs), to shuttle between tumor cells and TME, thereby affecting tumor development [34–37]. Among the biologically active substances, tumor-derived exosomal miRNAs can induce TME heterogeneity while changes in TME promote tumor progression. This paradigm, similar to a positive feedback loop, makes the uncontrollable growth of the tumor [38–43]. In this article we updated the interaction of exosomal miRNAs and TME.

## The overview of microenvironment and exosomes in cancer

### The components of tumor microenvironment

Growth, metastasis and treatment resistance of tumors are inseparable from the support of TME, a dynamic ecosystem containing multiple cell types and non-cellular components. Some of the basic biological behavioral features of tumors, such as proliferation, migration, invasion, apoptosis inhibition, immune evasion, angiogenesis, and metabolic reprogramming are all affected by TME. The complex communication network in TME is the basis for the regulation of these biological functions, including autocrine and paracrine. Exocrine-mediated communication is an important emerging pathway in paracrine signal transduction [2].

Non-tumor cells in TME, such as fibroblasts, endothelial cells and immune cells, are affected by tumor-related active substances, and their original cellular functions undergo tumor-like changes, constantly adapt to new environments and promote tumor growth. Due to the influence of TME, NFs are activated into CAFs. CAFs are the most abundant stromal cells in TME, producing an ECM that differs from normal ECM in terms of stiffness and alignment, which support tumor cells migration [9]. Hypoxia in TME causes tumor to secrete angiogenic factors to act on endothelial cells and promote angiogenesis [44, 45]. The immune cells in TME show diversity, and they block the immune response. The inflammatory molecules around the tumor cells also cause the system to fail to recognize and eliminate cancer cells [38, 46, 47]. These make TME a complex heterogeneous environment and often leads to an uncontrollable trend in the development of tumors [48, 49].

### The biosynthesis and function of extracellular vesicles and exosomes

Extracellular vesicles (EVs) are nano-sized lipid bilayer vesicles (40-1000nm in diameter) released by cells or detached from the plasma membrane [50, 51]. EVs are generally divided into two categories: ectosomes and exosomes. Ectosomes are vesicles formed from the plasma membrane sprouting outwards, including microvesicles, microparticles and large vesicles with a size range of 50-1000nm in diameter. Exosomes are small extracellular vesicle (sEVs) in a size range of 40-160nm in diameter with an endosomal origin. EVs have biological activities and mediate intercellular communication [36]. During tumor progression, EVs derived from different cells (tumor cells, stromal cells, immune cells, etc.) play an important role and participate in the formation of TME [44, 52–54].

In this review, we mainly focused on the exosomes. However, because of absence of strict standards for exosome isolation and purification methods, the International Society for Extracellular Vesicles encouraged researchers to establish minimum requirements and strictly control the integrity, size, molecular cargo, and functionality of the vesicle population [38, 55–57], so that we narrowed the research of exosomes based on the widely accepted methods. Exosomes are small extracellular vesicles (40-160 nm in diameter) formed by dynamic exocytosis [58–60]. Exosomes originate from the luminal cavity or early intracellular bodies in the circulation pathway of the plasma membrane. These membranes or early intracellular bodies will sag inward to form intraluminal vesicles (ILV), which will further develop into multivesicular bodies (MVB) [61, 62]. In general, multivesicular bodies are fused with lysosomes to be degraded, but some multivesicular bodies are fused to the cell surface under the traction of intracellular molecular motors and eventually secreted outside the cell, which called exosomes [36, 63].

Exosomes are involved in the biology of many diseases. Exosomes can regulate the immune response and inflammation, possibly through transfer and presentation of antigen peptides, to induce expression of inflammatory genes in recipient cells [64, 65]. In metabolism and cardiovascular diseases, exosomes induce metabolic disorders in adipocytes and islet cells [66, 67]. Exosomes may impair the formation of neurotoxic oligomers and promote neurodegeneration [68–70]. More importantly, exosomes are associated with tumor growth, angiogenesis, metastasis, sensitivity to chemotherapy, and immune evasion [47, 71, 72].

### miRNAs sorting to exosomes

Exosomes contain a variety of biologically active molecules, such as proteins, lipids and nucleic acids. miRNAs are one of them and play an important role in intercellular cellular transport and signal transduction [73–75]. Exosomes can transfer metabolites and promote communication between different cells through the exchange of exosomal miRNAs, and then play an immune response, tumor microenvironment remodeling and tumor metastasis during tumor progression [38, 76–78]. Many reports indicate that exosomes affect the biology of recipient cells by transferring miRNAs from donor cells to recipient cells, but the mechanism of how exosomes sorting miRNAs has not been thoroughly solved. According to exosomes database (www.exocarta.org), 2838 miRNAs are listed in the latest update. Among the 2588 annotated miRNAs in the human genome, 593 miRNAs have been detected in exosomes [79]. Four potential mechanisms for sorting miRNAs into exosomes were proposed. The neural sphingomyelinase 2 (nSMase2) was the first molecule found to be linked with miRNAs packaging into exosomes. Overexpression of nSMase2 leads to an increased number of miRNAs loaded into exosomes. This suggests that the neural sphingomyelinase 2 (nSMase2)-dependent pathway is associated with the sorting of exosomal miRNAs [80]. The second is based on the control of the sumoylated form of heterogeneous nuclear ribonucleoprotein (hnRNP). Sumoylated hnRNPA2B1 controls the sorting of exosomal miRNAs by recognizing the GGAG and GGCU base sequence of the 3 ’end region of miRNAs [81, 82]. The third is that most exosomal miRNAs isolated from urine or B cells were uridylated at 3 ′ ends. The sorting of miRNAs to ILV may also require hydrophobic modification and GGAG base sequence at 3 ′ end of the miRNAs. This indicates that the 3 ’ends of miRNAs may be involved in directing miRNAs into exosomes [83, 84]. Finally, there are reports that Argonaute proteins (functional carriers of miRNAs) are related to the selection of exosomal miRNAs. Knocking out Argonaute 2 (Ago2) reduces the contents of certain exosomal miRNAs, such as miR-142-3p, miR-150, and miR-451 [85, 86]. In summary, some specific protein complexes and miRNAs own structural characteristics have affects the miRNAs' transfer to exosomes, but the complete sorting mechanism and process have not yet been elucidated and need further exploration.

## The role of exosomal miRNAs in TME

During the progression of the tumor, primary tumor-derived exosomal miRNAs can be transferred to non-malignant cells in the tumor microenvironment to induce heterogeneity [50, 87–89]. At the same time, with the changes in biological activity of non-malignant cells in the tumor microenvironment, non-malignant cells can also secrete exosomal miRNAs to further regulate tumor cells or other microenvironmental components [40, 90]. In most studies, the stromal cell receptors of cancer-derived exosomal miRNAs are cancer-associated fibroblasts (CAFs), endothelial cells and immune cells dynamically regulate each other in TME. Exosomal miRNAs on the heterogeneity of TME is mainly reflected in the fact that exosomal miRNAs can activate cancer-associated fibroblasts and thus reshape ECM, which is beneficial to the spread of tumor cells. Exosomal miRNAs promote endothelial cells to form tubes, and the formation of abundant vascular networks is conducive to the metabolism and survival of tumor cells. Exosomal miRNAs also mediate inflammatory cell infiltration and immune escape, which is conducive to colonization and proliferation of tumor cells. Through these macroscopic effects, exosomal miRNAs can make TME more suitable for tumor development [91]. Herein, we focused on the roles of exosomal miRNAs in following aspects.

### Reshaping ECM to promote tumor progression

Extracellular matrix (ECM) is composed of protein and carbohydrates, with the functions of connection, support, water retention, anti-stress and protection. ECM supports the basic life activities of cells, such as proliferation, differentiation, and migration [92, 93]. However, tumors are often accompanied by dysfunction of ECM. Tumor development is a complex process involving dynamic interactions between malignant cells and their surrounding stroma composed of cells and non-cellular components. Within the stromal, fibroblasts represent not only the major cell types, but also the main source of extracellular matrix (ECM) and soluble factors [94, 95]. Normal fibroblasts exert multiple inhibitory functions against cancer-initiating and metastasis through direct cell-cell contact, paracrine signaling, and ECM integrity [96]. However, tumor-derived exosomal miRNAs can trigger a series of tumor-promoting signals, inducing normal fibroblasts (NFs) transformation into CAFs, which changes the original ECM physiological state, thus creating the optimal niche for the widespread growth of cancer cells [96, 97].

In tumors, tumor cell-derived exosomal miRNAs are highly diverse and are capable of differentiating NFs into CAFs through a variety of signaling pathways. Exosomal miRNAs from cancer cells elicit a parenchymal signaling response at the receptor site and effectively inducing fibroblast activation, such as α-smooth muscle actin (α-SMA), fibroblast growth factor 2 (FGF2) and fibroblast activating protein (FAP) expression [98–100]. Matrix composed of CAFs is conducive to the proliferation and migration of tumor cells. In ovarian cancer, the cancer-related exosomal miR-124 targets sphingosine kinase 1 (SPHK1) and upregulates α-SMA and FAP, which differentiates NFs into CAFs and regulates CAFs migration and invasion [101, 102]. High expression of exosomal miR-27b-3p and miR-214-3p in myeloma cells triggers proliferation and apoptotic resistance of bone marrow fibroblasts via the FBXW7 and PTEN/AKT/GSK3 pathways. At the same time, miR-27-3p and miR-214-3p were up-regulated in fibroblasts co-cultured with myeloma, and activated expression of fibroblast activation markers α-SMA and FAP. The biological behavior of bone marrow fibroblasts is programmed to alter the myeloma microenvironment [103, 104]. Exosomal miRNAs in digestive system tumors also reshaped ECM in adjacent sites and promote tumor progression. Exosomal miR-27a derived from gastric cancer (GC) is transported to fibroblasts, and thus results in decreased expression of CSRP2, enhanced expression of α-SMA, and differentiation of fibroblasts into CAFs [105]. Exosomal miR-10b secreted by colorectal cancer cells can be transferred to fibroblasts, where it inhibits PIK3CA expression and PI3K/Akt/mTOR pathway activity, promote expression of TGF-β and α-SMA, and enable fibroblasts to acquire the characteristics of CAFs [106, 107]. These changes promote the proliferation, migration and invasion of tumor cells. Exosomal miRNAs have found similar effects in colorectal cancer (CRC). Exosomal miR-2149-5p, miR-6737-5p, and miR-6819-5p can inhibit the expression of TP53 in fibroblasts to promote tumor proliferation [108].

**Fig. 1.**
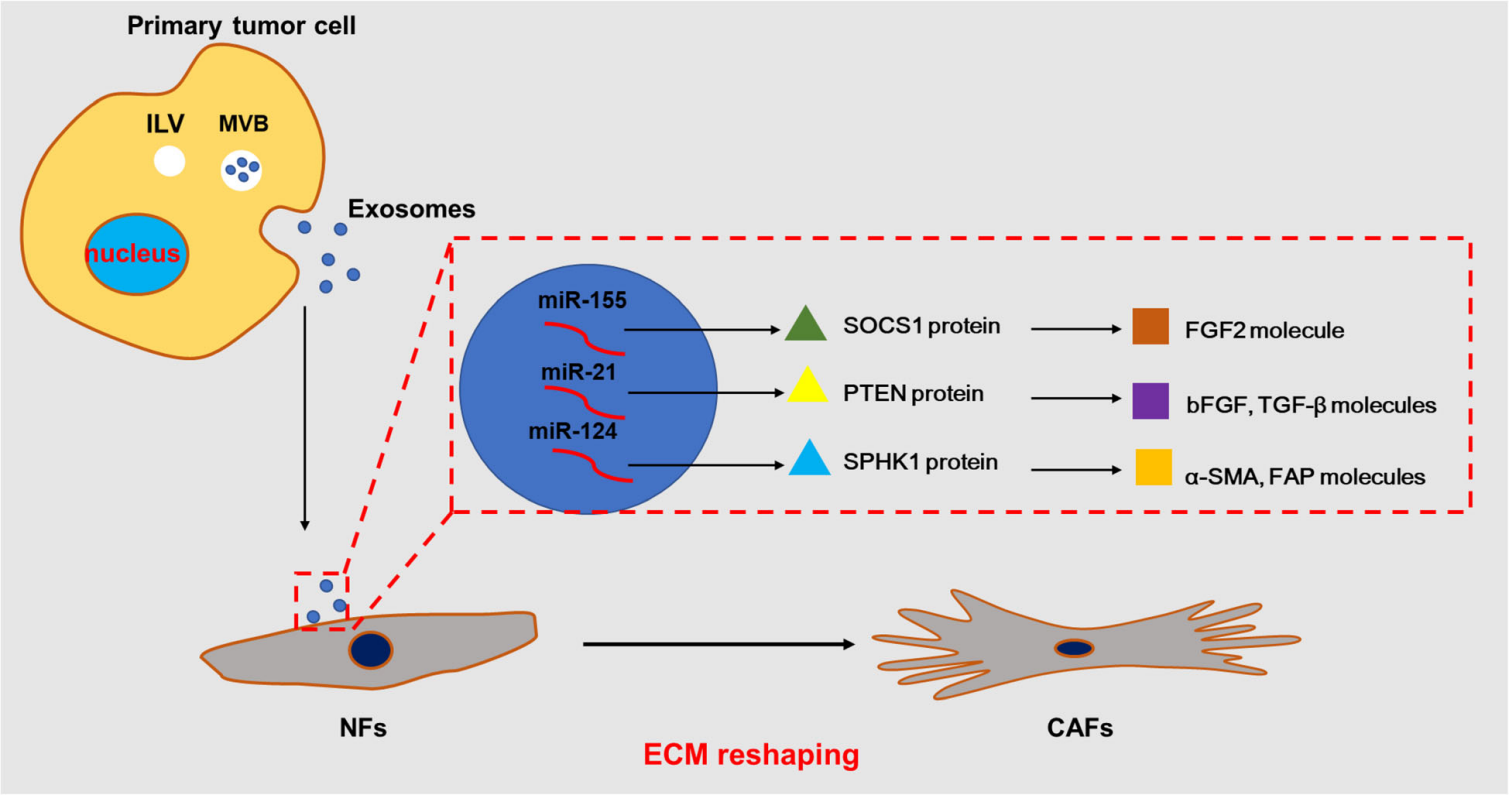
The mechanism of ECM reshaping by exosomal miRNAs . Exosomes secreted by the primary tumor cells are taken up by the receptor NFs, wherein the exosomal miRNAs (miR-155, miR-21, miR-124, etc.) target the proteins (SOCS1, PTEN, SPHK1, etc.) and activate the molecules (FGF2, bFGF, TGF-β, α-SMA, FAP, etc.). These exosomal miRNAs reshape the ECM by inducing the conversion of normal fibroblasts (NFs) into cancer-associated fibroblasts (CAFs).

In addition, changes in ECM also affect angiogenesis, inflammatory response, and metabolic reprogramming. Phenomenon was shown in melanoma where highly expressed exosomal miR-155 inhibits the expression of SOCS1, activates the JAK2/STAT3 pathway, up-regulates the expression of FGF2, VEGFA and MMP9 in CAFs, and promotes the formation of blood vessels in the tumor [109, 110]. In hepatocarcinoma (HCC), exosomal miR-21 is transferred to CAFs, directly targeting PTEN to activate PDK1/Akt signaling, up-regulating VEGF, MMP2, MMP9, bFGF, and TGF-beta and thus promoting angiogenesis [111, 112]. Exosomal miR-1247 targets B4GALT3 and activates the beta1-integrin-NF-kappaB signaling pathway, which activates CAFs to secrete the inflammatory cytokines IL-6 and IL-8 and induce inflammatory infiltration [113]. Exosomal miR-9 and miR-105 are derived from breast cancer; the former promotes the activation of NFs into CAFs by affecting MMP1, EFEMP1 and COL1A1 [30], and the latter activates MYC signal transduction to induce metabolic reprogramming of CAFs, and adapts CAFs to different metabolic environments, promoting tumor growth [18]. Similar reports include miRNA-142-3p in EVs secreted by lung cancer cells, which promotes the cancer phenotype of lung fibroblasts [114] (Fig. 1 and Table 1).

**Table 1:** Exosomal miRNAs involved in tumor microenvironments

Host cell	Exosomal miRNAs	Target mRNA	Involved meleculs	Function	Reference
OC	miR-124	SPHK1	α-SMA, FAP	Reshaping ECM	[101, 102]
Myeloma	miR-27-3p, miR-214-3p	FBXW7	α-SMA, FAP	Reshaping ECM	[103, 104]
GC	miR-27a	CSRP2	α-SMA	Reshaping ECM	[105]
CRC	miR-10b	PIK3CA	α-SMA, TGF-β	Reshaping ECM	[106, 107]
CRC	miR-2149-5p, miR-6737-5p, miR-6819-5p		TP53	Reshaping ECM	[108]
Melanoma	miR-155	SOCS1	FGF2	Reshaping ECM	[109, 110]
HCC	miR-21	PTEN	bFGF, TGF-β	Reshaping ECM	[111, 112]
HCC	miR-1247	B4GALT3	β1-integrin-NF-κB	Reshaping ECM	[113]
Breast cancer	miR-9		MMP1, EFEMP1, COL1A1	Reshaping ECM	[30]
Breast cancer	miR-105		MYC	Reshaping ECM	[18]
CAFs	miR-148b	DNMT1	EMT-related molecules	Promoting metastasis	[65]
CAFs	miR-196a	CDKN1B, ING5	P21, CDK2, CDK4, Cyclin D1 and Cyclin E1	Chemotherapy resistance	[115]
CAFs	miR-522		ALOX15	Chemotherapy resistance	[12]
NPC	miR-23a	TSGA10	p-ERK	Promoting angiogenesis	[121, 122]
Glioma	miR-21		VEGF, p-FLK, VEGFR2	Promoting angiogenesis	[123, 124]
HCC	miR-210-3p	SAMD4, STAT6		Promoting angiogenesis	[125]
MSCs	miR-100		mTOR, HIF-1α, VEGF	Promoting angiogenesis	[127]
NSCLC	miR-619-5p		RCAN1.4	Promoting angiogenesis	[128]
OC	miR-205	PTEN	p-AKT, p-ERK	Promoting angiogenesis	[129]
CRC	miR-25-3p	KLF2	VEGFR2, p-AKT, p-ERK	Promoting angiogenesis	[130]
CRC	miR-25-3p	KLF4	ZO-1, Occludin, Claudin5	Promoting angiogenesis	[130]
Lung cancer	miR-23a	PHD1, PHD2, ZO-1	HIF-1α	Promoting angiogenesis	[132]
Glioma	miR-9	MYC, OCT4	MYC, OCT4	Promoting angiogenesis	[133]
HCC	miR-451	LPIN1		Inhibiting angiogenesis	[134]
NPC	miR-9	MDK	PDK, AKT	Inhibiting angiogenesis	[135]
Pancreatic cancer	miR-212-3p	RFXAP	HLA-DR, -DP, -DQ molecules	Suppressing immune	[145, 146]
Pancreatic cancer	miR-203	TLR4, TNF-α, IL-12	TLR4, TNF-α, IL-12	Suppressing immune	[147]
Breast cancer	miR-let-7i		TGF-β, IFNγ, TLR4	Suppressing immune	[148]
Treg	miR-150-5p, miR-142-3p	IL-10, IL-6	IL-10, IL-6	ISuppressing immune	[149]
Treg	miR-let-7d		IFNγ	Suppressing immune	[150]
NSCLC	miR-125b		p53	Enhancing immune	[154]
Melanoma	miR-125b-5p	LIPA	Il-1β, CCL1, CCL2, CD80	Enhancing immune	[71]
Head and neck cancer	miR-21	MRC1	MRC1	Suppressing immune	[155]
EOC	miR-222-3p	SOCS3	p-STAT3	Suppressing immune	[156]
EOC	miR-21-3p, miR-125b-5p, miR-181d-5p, miR-940			Suppressing immune	[157, 158]
Pancreatic cancer	miR-301a-3p	PTEN	p-mTOR, p-AKT, PI3K p110γ	Suppressing immune	[159, 160]
Colon cancer	miR-1246		IL-10, TGF-β, MMPs	Suppressing immune	[161]
Glioma	miR-10a	RORA	p53	Suppressing immune	[165]
Glioma	miR-21	PTEN	p-STAT3, p-p65, p-AKT	Suppressing immune	[165]
CLL	miR-155		p-STAT1, NF-κ	Suppressing immune	[166]
MDSCs	miR-126a		IL-13, IL-33	Promoting angiogenesis	[167]

These researches show that cancer-derived exosomal miRNAs can affect the physiological function of stroma. Conversely, a reciprocal exosomal miRNAs exchange from the stroma to cancer cells also modulates cancer progression. For example, CAFs-derived exosomal miR-148b in the matrix surrounding endometrial cancer can up-regulate DNMT1, leading to changes in EMT-related molecules like E-cadherin, N-cadherin, vimentin, and fibronectin and promoting cancer cell metastasis [65]. CAFs are resistant to cisplatin and deliver exosomal miR-196a, which binds to target CDKN1B and ING5, mediates the expressions of p27, CDK2, CDK4, Cyclin D1 and Cyclin E1 and thus induces cisplatin resistance to cancer cells [115]. CAFs-derived exosomal miR-522 reduces the contents of lipid-ROS in gastric cancer cells by inhibiting the expression of ALOX15, which leads to a decrease in the sensitivity of gastric cancer to chemotherapy [12].

Compared with NFs, CAFs have the characteristics of excessive proliferation and unique cytokines. This not only induces the formation of new blood vessels, but also promotes the entry of immune cells into TME, which greatly changes the physiological function of ECM to support tumor proliferation, metastasis and treatment resistance [116, 117]. However, cells involved in ECM formation are not only fibroblasts, but also chondrocytes, osteoblasts, and certain epithelial cells. Exosomal miRNA remodeling of ECM can also be achieved by affecting the function of these cells. For example, studies have shown that cancer-secreted exosomal miR-940 promotes osteogenic differentiation of mesenchymal cells by targeting ARHGAP1 and FAM134A, and then induces osteogenic phenotypes in the bone metastasis microenvironment and promotes tumor metastasis [118]. But research on the interaction of exosomal miRNAs with these cells is not comprehensive. At the same time, the composition of ECM not only includes collagen (synthesized by fibroblasts, chondrocytes, osteoblasts and certain epithelial cells and secreted outside the cell), but also includes non-collagen glycoproteins, glycans and elastin. Whether exosomal miRNAs reshape ECM by affecting these ingredients remains to be proven.

### Promoting angiogenesis to enhance proliferation and migration

Tumor growth depends to a large extent on the metabolism of cancer cells [119]. The disordered distribution of tumor blood vessels and the loss of normal vascular function lead to local hypoxia and impaired nutrient supplies. At the same time, the distance gradient between different vascular beds also leads to the imbalance of drug distribution and absorption [120]. These changes of vascular network promote the formation of internal microenvironment and intratumoral heterogeneity.

The exosomal miRNAs can be taken up by the vascular endothelial cells to change the original distribution and physiological functions of the blood vessels in the microenvironment. Exosomal miRNAs secreted by tumor cells have been reported to promote angiogenesis in TME. In nasopharyngeal carcinoma (NPC), exosomal miR-23a mediates angiogenesis by repressing TSGA10 and phosphorylation of ERK, which enhances tube generation ability of human umbilical vein endothelial cells (HUVECs) *in vitro* and *in vivo* [121, 122]. Glioma stem cell-derived exosomal miR-21 stimulates VEGF/p-FLK/VEGFR2 signaling pathway to promote angiogenesis in endothelial cells [123, 124]. The exosomal miR-210-3p secreted by HCC cells is transferred to endothelial cells, targeting SMAD4 and STAT6 to promote angiogenesis, and it is found that the higher miR-210-3p in the serum of HCC patients is positively correlated with the microvessel density in HCC tissues [125]. EVs and sEVs-mediated miRNAs transfer also promotes angiogenesis in TME. In NSCLC, EVs-mediated miR-142-3p transferred to endothelial cells and fibroblasts, inhibiting the expression of TGFβR1, PDGFR-β and p-SMAD2/3 to promote angiogenesis [114]. Human ovarian carcinoma cell line SKOV-3 secretes miR-141-3p in small extracellular vesicles (sEVs), which activates the JAK-STAT3 pathway in endothelial cells and promotes angiogenesis [126]. Besides, exosomal miRNAs that promote angiogenesis can also be derived from other cells. Exosomal miR-100 from human mesenchymal stem cells (MSCs) affects the mTOR/HIF-1α/VEGF signaling axis to promote angiogenesis in breast cancer [127].

**Fig. 2.**
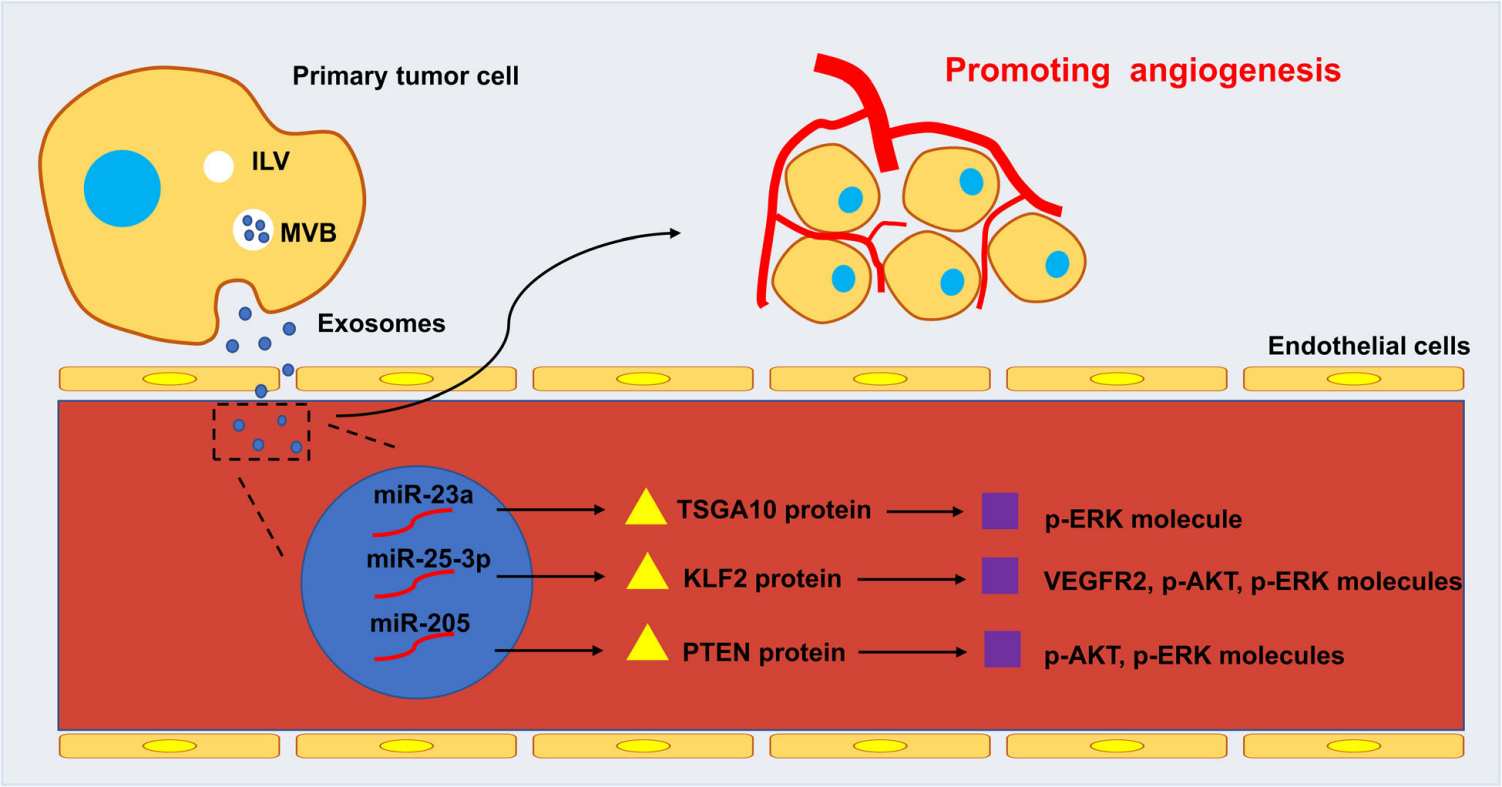
The mechanism of angiogenesis promoted by exosomal miRNAs. Exosomes secreted by the primary tumor cells are taken up by the receptor endothelial cells, wherein the exosomal miRNAs (miR-23a, miR-25-3p, miR-205, etc.) target the proteins (TSGA10, KLF2, PTEN, etc.) and activate the molecules (VEGFR2, p-AKT, p-ERK, etc.). These exosomal miRNAs promote angiogenesis by regulating the number of local blood vessels and physiological functions.

The rich vascular network in TME is beneficial to the proliferation and metastasis of cancer cells. Exosomal miR-619-5p inhibits the expression of RCAN1.4, promotes angiogenesis, and facilitates the growth and metastasis of cancer cells [128]. Recent studies have shown that circulating exosomal miR-205 expression is elevated in OC patients and is related to microvessel density, and exosomal miR-205 induces angiogenesis via the PTEN-AKT pathway, and promotes tumor cell proliferation *in vitro* [129]. Changes in the vascular microenvironment are not only in the number of blood vessels, but also in vascular permeability, adhesion, and ability to form a ring. The colorectal cancer-derived exosomal miR-25-3p can down-regulate KLF2 and KLF4, and KLF2 affects the tube formation ability of HUVECs through the VEGFR2/p-Erk/p-Akt pathway while KLF4 activates ZO-1/Occludin/Claudin5 pathway to affect the growth of the aortic rings, which in turn changes the vascular microenvironment [130, 131]. Under hypoxic conditions, lung cancer cell-derived exosomal miR-23a directly inhibits prolyl hydroxylase 1 and 2 (PHD1 and PHD2) and accumulates HIF-1α in endothelial cells, inducing angiogenesis, and exosomal miR- 23a also inhibits ZO-1, increasing vascular permeability and transendothelial migration of cancer cells [132]. In human glioma, exosomal miR-9 promotes angiogenesis, vascular permeability and adhesion through the MYC/OCT4 pathway [133] (Fig. 2**)**.

Exosomal miRNAs influence on vascular network is not only promotion, but sometimes also play an inhibitory effect. Studies have found that exosomal miR-451 acts as a tumor suppressor and targets LPIN1 to induce apoptosis both in HCC cell lines and HUVECs. In addition, miR-451a suppresses HUVECs tube formation and vascular permeability [134]. NPC-derived exosomal miR-9 up-regulates MDK and activates the PDK/Akt signaling pathway to inhibit the formation of endothelial cells. High expression of MDK in NPC tumor samples is positively correlated with microvessel density, revealing the anti-angiogenic effects of exosomal miR-9 in the development of nasopharyngeal carcinoma [135]. Except for tumor-derived exosomal miRNAs, which inhibit angiogenesis, non-tumor cells have similar functions. miR-15a, miR-181b, miR-320c, and miR-874 in EVs released by human liver stem-like cells (HLSCs) possess an anti-tumorigenic effect by inhibiting tumor angiogenesis [136]. According to these reports, it can be found that exosomal miRNAs can regulate the vascular network in TME through multiple signaling pathways, but these molecular mechanisms have not been fully elucidated and need to be explored in the future.

### Promoting the formation of immunosuppressive environment

In the TME, immune cells including lymphocytes, dendritic cells, and macrophages, regularly infiltrate tumor tissues and adjacent sites. Through multiple signal transduction pathways mediated by exosomal miRNAs, tumor cells can inhibit the maturation and differentiation of immune cells, thereby creating an immune microenvironment suitable for tumor growth [41, 137, 138]. At the same time, in hypoxia and low nutrient supplies in the microenvironment, tumor cells often secrete metabolic by-products, such as lactic acid, nitric oxide, reactive oxygen species, prostaglandins and arachidonic acid, leading to the formation of an inflammatory microenvironment [139, 140]. Changes in the biological functions of various immune cells in microenvironment and the production of inflammatory mediators result in tumor cell escaping from immune surveillance.

Dendritic cells (DCs) are the most powerful professional antigen presenting cells in the body. Mature DCs can effectively activate the initial T cells and maintain the central part of the immune response [141, 142]. Tumor-derived and endogenous exosomal miRNAs can regulate cross-presentation in dendritic cells and with other immune cells, this exomsomal miRNAs-mediated intercellular communication may affect the maturation of DCs [143, 144]. In pancreatic cancer, exosomal miR-212-3p targets MHC class II TF RFXAP resulting in reduced expression of HLA-DR, -DP, and -DQ molecules and thus interfering with the function of DCs cells [145, 146]. Exosomal miR-203 is able to reduce the expression of TLR4, TNF-α and IL-12 in DCs, affecting the activation of natural killer cells (NKs) [147]. Up-regulated exosomal miR-let-7i in tumor-derived exosomes (TEX) can be taken up by mDCs, resulting in changes in intracellular levels of IL-6, IL-17, IL-1b, TGFbeta, SOCS1, KLRK1, IFNγ, and TLR4, thereby suppressing the immune response [148]. miRNAs from regulatory T cells (Treg) can also affect the immune response, EVs-mediated miR-150-5p and miR-142-3p can be transferred to DCs to induce a cell-refractory phenotype, resulting in increased IL-10 and decreased IL-6 expression [149], exosomal miR-let-7d is transferred to T helper 1 (Th1) cells to inhibit Th1 cells proliferation and IFNγ secretion, and IFNγ secreted by Th1 cells (a subtype of Naïve CD4 T cells) plays a central role in anti-tumor immunity [150].

**Fig. 3.**
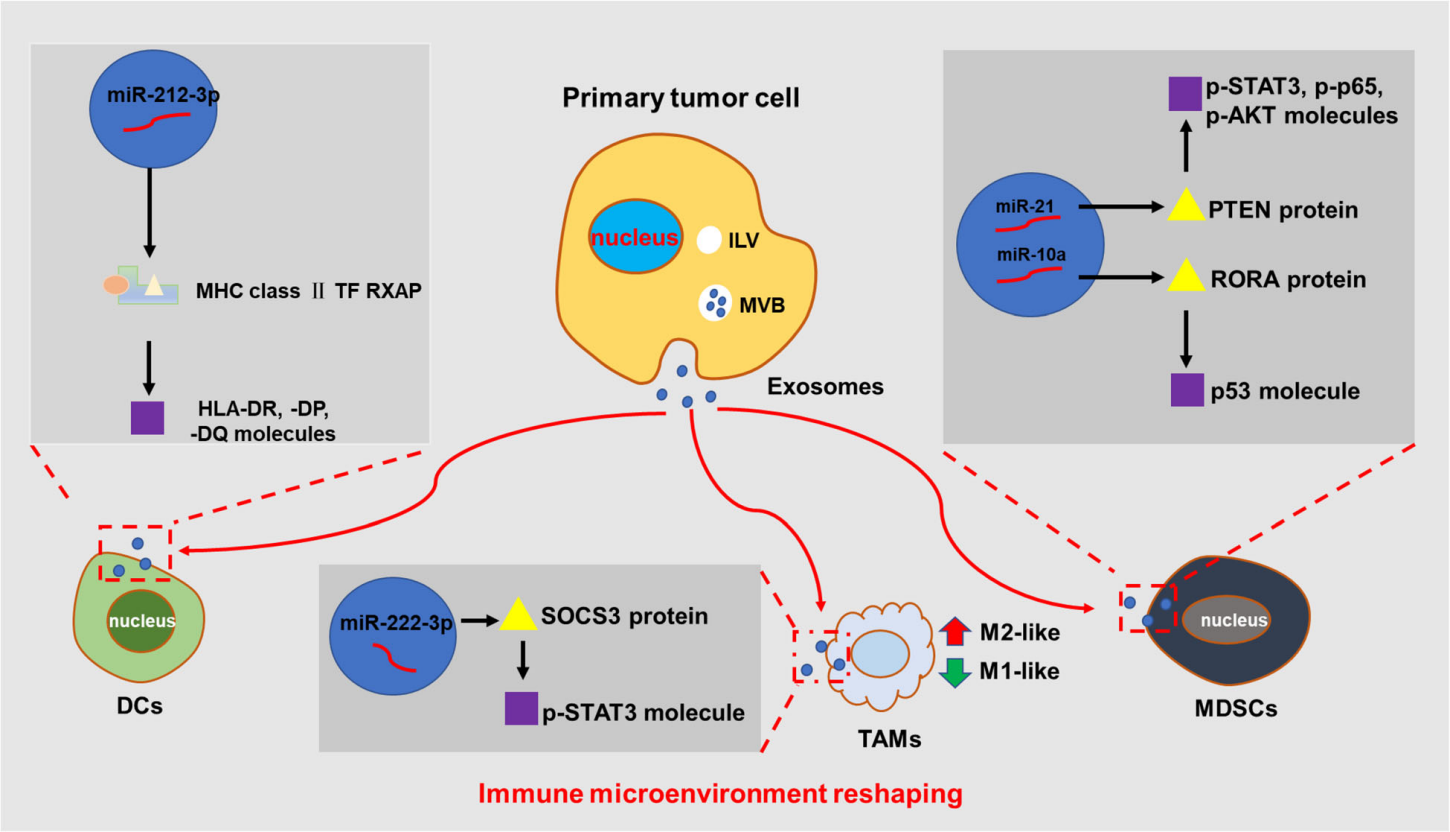
The mechanism of immune microenvironment reshaping by exosomal miRNAs. Exosomes secreted by the primary tumor cells are taken up by the receptor immune cells, wherein the exosomal miR-212-3p target the MHC class II TF RFXAP complexus and activate the HLA-DR, -DP, and -DQ molecules. The exosomal miRNAs (miR-222-3p, miR-21, miR-10a, etc.) target the proteins (SOCS3, PTEN, RORA, etc.) and activate the molecules (p-STAT3, p-p65, p-AKT, p53, etc.). These exosomal miRNAs reshape inmune microenvironment by mediating immunosuppression.

Tumor-associated macrophages (TAMs) are one of the most abundant immune cells in TME. TAMs play a huge role in the proliferation and migration of tumor cells and counteract the cytotoxic effect of T lymphocytes and NKs, facilitating cancer cells to evade immune surveillance [140, 151]. TAMs have strong plasticity and can differentiate into immune-stimulating (M1-polarized) TAMs or oppositely immune-suppressive (M2-polarized) TAMs, respectively, having different biological functions [152]. TAMs in tumors often behave as M2 phenotype and are usually associated with poor prognosis [153]. A large number of studies have reported that exosomal miRNAs can regulate the phenotypes of TAMs. Exosomal miR-125b derivied from lung adenocarcinoma cells promotes macrophage repolarization toward an anti-tumor M1 phenotype [154]. Exsomal miR-125b-5p secreted by melanoma cells targets LIPA and increases the expression of M1 phenotype markers IL-1β, CCL1, CCL2, and CD80 [71]. Oppositely, exosomal miR-21 taken up by CD14^+^ human monocytes inhibits the expression of the M1 marker and increases the expression of the M2 marker. Knockout of miR-21 in Snail-expressing human head and neck cancer cells attenuated M2 polarization of TAMs, and miR-21 was found to be positively correlated with M2 marker MRC1 in head and neck cancer tissues [155]. In epithelial ovarian cancer (EOC), exosomal miR-222-3p can be transferred to macrophages, down-regulating SOCS3, inducing phosphorylation of STAT3, and thus leading to polarization of the M2 macrophages [156]. In hypoxia, EOC-derived exosomal miR-21-3p, miR-125b-5p, miR-181d-5p, and miR-940 differentiate TAMs into M2 phenotypes and promote tumor progression [157, 158]. Likewise, exosomal miR-301a-3p derived from hypoxic pancreatic cancer cells activates the PTEN/PI3Kγ signaling pathway to trigger M2 phenotype polarization of macrophages [159, 160]. Mutant p53 colon cancer cells-derievd exosomal miR-1246 induces M2 polarization of macrophages and reshapes the TME through increase the expression of IL-10, TGFβ, and MMPs [161] **(Fig.**3**)**.

Abnormal differentiation and function of myeloid cells is a hallmark of cancer. Among them, myeloid-derived suppressor cells (MDSCs) have the function of suppressing adaptive immunity and innate immune response, and play an important role in tumor immune escape [162–164]. Exosomal miRNAs affect the function of MDSCs by regulating the activity of transcription factors and transcription activators, thereby reshaping the immune microenvironment. In the research of glioblastoma, exosomal miR-10a targets RORA and affects the differentiation of MDSCs through the NFκB pathway, exosomal miR-21 targets PTEN and affects the activation of MDSCs via the p-STAT3/p-p65/p-Akt pathway [165]. Exosomal miR-155 istransmitted to monocytes, leading to nuclear translocation of NFkB and phosphorylation of STAT1, reprograming conventional monocytes into MDSCs [166]. Changes in the function of MDSCs affect the progression of the tumor itself. Recent research shows that exosmal miR-126a derived from MDSCs promotes angiogenesis and benefit breast cancer lung metastases [167].

The immunomodulation induced by exosomal miRNAs is complex and dynamic. In TME, tumor cells interact with various types of immune cells and cross-promote immunosuppressive activity. Among them, exosomal miRNAs play a pivotal role in them, but the mechanism has not been elucidated. Thereby, the function of exosomal miRNAs in the reciprocal interplays between cancer cells and hosts immune system merits further investigation.

## Perspectives of exosomal miRNAs

With the vigorous development of the biology of exosomes in tumors, more and more evidence indicates that exosomal miRNAs play an important role in tumor progression and TME reshaping. Compared with miRNAs released directly into the circulatory system, exosomal miRNAs are protected by lipid bilayer encapsulation and avoid degradation by ribonuclease in the blood. Notebalely, exosomal miRNAs are more bioactive pool of circulating miRNAs compared to those miRNAs transported with liposomes [41, 168, 169]. Considering the advantages of exosomal miRNAs and the widespread presence of exosomes in all biological fluids (blood, breast milk, semen, and urine), diagnostic and therapeutic technologies based on exosomal miRNAs have a bright future.

Some specific exosomal miRNAs have high diagnostic value in tumors, and detecting them is helpful for early diagnosis of tumors. For example, in prostate cancer, breast cancer, and oral squamous cell carcinoma, the expression of exosomal miR-1246 is closely related to pathological grades, distant metastasis and poor prognosis [170–173]. Circulating exosomal miR-375 is valuable for the diagnosis of ovarian, rectal and prostate cancer [174–176]. The combination of multiple exosomal ncRNAs can enhance the diagnostic and prognostic potential of exosomal miRNAs. For example, the combination of expression of plasma exosomal miR-30d-5p and let-7d-3p is valuable diagnostic markers for non-invasive screening of cervical cancer and its precursors [177]. Circulating exosomal miRNA-21 and lncRNA-ATB are related to the TNM stage of liver cancer and other prognostic factors, including the T stage and portal vein thrombosis [178].

Exosomal miRNAs, as a new tumor treatment method, are being widely explored. Based on the fact that exosomal miRNAs effectively bind to target mRNA and inhibit gene expression in recipient cells, related exosomal engineering techniques have been used to treat tumors by delivering tumor suppressor exosomal miRNAs. For example, delivery of exogenous miR-155 into DCs using TEX as a vector results in increased expression of MHCII (I/A-I/E), CD86, CD40 and CD83, promoting activation of DCs. Exosomal miRNA-155 significantly increases the levels of IL12p70, IFN-γ and IL10 and improves immune function [179]. By fusing Her2 affinity to the extracellular N-terminus of human Lamp2, and then using the modified exosomes to co-deliver 5-FU and miR-21 inhibitors (miR-21i), which targets colon cancer cells, effectively reverses the resistance of tumor cells and significantly enhances the toxicity of 5-FU resistant cancer cells [180].

Although exosomal miRNAs have made exciting progress in oncology, most of these results are experimental. Extension of exosomal miRNAs technologies to clinic remains challenging. There is no doubt that the function of exosomes is determined by their specific contents. A large amount of literature has reported that tumor-derived exosomal miRNAs can reshape TME and promote tumor progression, but little is known about the sorting mechanism of exosomal miRNAs. Although the basic framework of the endosome sorting complex required for transport (ESCRT) and Ago2 in MVB sorting has been reported in previous studies, it remains to be elucidate whether other novel sorting signals are involved in the release of exosomal miRNAs [86, 181–184].

The potential of exosomal miRNAs as diagnostic markers is unquestionable, but how to improve the sensitivity and specificity of exosomal miRNAs remains to be solved. The combination of different exosomal cargos, such as proteins, lipids, RNA and miRNAs for cancer diagnosis and prognosis can more comprehensively reflect the characteristics of tumors. At the same time, the scope of application of exosomal miRNAs also needs attention. The expression level of exosomal miRNAs is related to tumor types, clinical stages or other underlying diseases, and there are differences between individual patients. Therefore, how to use exosomal miRNAs accurately is also worth of considering.

The widespread use of exosomal miRNAs in clinical treatment remains challenging. First, exosomes-based therapeutic tools require more accurate and standardized exosomal purification methods, and the economic cost of mass-producing exsomes for clinical application cannot be ignored [56, 57]. The second is that exosomal miRNAs-induced biological behavioral changes are often released through the cultivation of supra-physiological numbers of cell, and how many orders of magnitude of exosomal miRNAs are needed to achieve the corresponding efficacy in clinical applications remains to be determined.

## Conclusion

Exosomal miRNAs, as a signaling molecule for communication between tumor cells and TME, play an important role in the formation and remodeling of TME, but its regulatory mechanism is still worth of further exploration. At present, most of the biological studies of exosomal miRNAs have been revealed by cell-culture systems *in vitro*. But the problems still remain whether exosomal miRNAs derived from supra-physiological numbers of cell reflect the biological conditions *in vivo*. It is necessary to conduct more experiments *in vivo* or in mammals.

With the increase of exosomes researches, people have gradually discovered that the exosomes obtained by traditional exosomal separation and purification methods (ultracentrifugation, density-gradient centrifugation, immune-affinity capture, and precipitation) not only contain sEVs, but also contain non-membrane structure vesicles (NVs). Components, double-stranded DNA (dsDNA) and histones, are more in the NVs rather than in exosomes or sEVs. Moreover, many of the most abundant miRNAs were more associated with extracellular NV fractions than with either parental cells or sEV fractions [56]. This indicates that we may need to re-evaluate the composition of exosomes, and it is urgent to explore the generation and sorting mechanisms of exosomal miRNAs or miRNAs in other type of sEVs.

## Data Availability

Not applicable.

## Abbreviations

TME: Tumor microenvironment
CAFs: Cancer-associated fibroblasts;
ECM: Extracellular matrix
MMPs: Matrix metalloproteinases
HIF-1α: Hypoxia inducible factor-1α
VEGF: Vascular endothelial growth factor
miRNAs: MicroRNAs
NFs: Normal fibroblasts
EVs: Extracellular vesicles
sEVs: Small extracellular vesicle
ILV: Intraluminal vesicles
MVB: Multivesicular bodies
nSMase2: Neural Sphingomyelinase 2
hnRNP: Heterogeneous nuclear Ribonucleoprotein
Ago2: Argonaute 2
α-SMA: α-Smooth muscle actin
FGF2: Fibroblast growth factor 2
FAP: Fibroblast activating protein
SPHK1: Sphingosine kinase 1
CRC: Colorectal cancer
HCC: Hepatocarcinoma
NPC: Nasopharyngeal carcinoma
HUVECs: Human umbilical vein endothelial cells
MSCs: Mesenchymal stem cells
PHD1 and PHD2: Prolyl hydroxylase 1 and 2
HLSCs: Human liver stem-like cells
DCs: Dendritic cells
NKs: Natural killer cells
Treg: Regulatory T cells
TAMs: Tumor-associated macrophages
EOC: Epithelial ovarian cancer
MDSCs: Myeloid-derived suppressor cells
ESCRT: Endosome sorting complex required for transport
NVs: Non-membrane structure vesicles
dsDNA: Double-stranded DNA
GC: Gastric cancer
NCSLC: Non-small cell lung cancer
CLL: Chronic lymphocytic leukemia.
